# Impact of Prior Selinexor Exposure on Outcomes of Chimeric Antigen Receptor T-Cell Therapy for Relapsed/Refractory Multiple Myeloma: An Exploratory Analysis

**DOI:** 10.3390/jcm14041316

**Published:** 2025-02-16

**Authors:** Bruno Almeida Costa, Danai Dima, Tomer Mark, Norah Layla Sadek, Stephen Ijioma, David Ray, Utkarsh Goel, George Dranitsaris, Tianxiang Sheng, Erin Moshier, Tarek H. Mouhieddine, Jack Khouri, Adriana Rossi

**Affiliations:** 1 Division of Hematology/Oncology, The Tisch Cancer Institute, Icahn School of Medicine at Mount Sinai, New York, NY 10029, USA; bruno.costa@mountsinai.org (B.A.C.); norah.sadek@mountsinai.org (N.L.S.);; 2 Fred Hutchinson Cancer Center, Seattle, WA 98109, USA; 3 Karyopharm Therapeutics, Newton, MA 02459, USA; 4 Department of Hematology and Medical Oncology, Taussig Cancer Center, Cleveland Clinic, Cleveland, OH 44106, USA; 5 Department of Public Health, Syracuse University, Syracuse, NY 13244, USA; george@augmentium.com

**Keywords:** multiple myeloma, CAR-T, selinexor, XPO1, T-cell fitness, T-cell exhaustion

## Abstract

**Background/Objectives**: Chimeric antigen receptor T-cell therapy (CAR-T) has become a key treatment option for relapsed/refractory multiple myeloma (RRMM), but factors impairing T-cell fitness may diminish efficacy. Our exploratory analysis aimed to evaluate the impact of prior treatment with a selinexor-containing regimen on CAR-T outcomes for RRMM patients. **Methods**: Data for this retrospective cohort study were sourced from electronic medical records at two US academic centers. Kaplan–Meier estimates assessed duration of response (DOR), progression-free survival (PFS), and overall survival (OS), reported as medians with interquartile ranges (IQRs). Cox proportional hazards regression analyzed factors potentially associated with PFS and OS, reported as hazard ratios (HRs) with 95% confidence intervals (CIs). **Results**: Among 45 patients exposed to selinexor before undergoing BCMA-directed CAR-T, median therapy line numbers for selinexor use and CAR-T were 7 and 9, respectively, with 24.4% receiving selinexor as part of bridging. At median follow-up of 68 months, median PFS and OS post CAR-T were 8.0 (IQR 3.1–39.5) and 35.9 (IQR 14.2–NR) months, respectively. Overall response rate to CAR-T was 89%, with a median DOR of 8.1 months (IQR 2.9–39.0). In our multivariable model, patients who received a selinexor-based regimen in the line of therapy preceding CAR-T showed a trend toward reduced risk of death (HR = 0.08; 95% CI 0.02–0.46) and/or disease progression (HR = 0.40; 95% CI 0.14–1.09). **Conclusions**: Prior selinexor exposure does not appear to compromise CAR-T outcomes in heavily pretreated RRMM, suggesting potential T-cell sparing. Our findings warrant larger, prospective studies to determine whether preemptive selinexor treatment can optimize CAR-T efficacy.

## 1. Introduction

The introduction of B-cell maturation antigen (BCMA)-directed chimeric antigen receptor T-cell therapy (CAR-T) in clinical practice has radically transformed the treatment paradigm of multiple myeloma (MM). Based on results of the KarMMa and CARTITUDE trials [1,2], two commercial products are currently approved for routine use in patients with both early and later lines of treatment: idecabtagene vicleucel (ide-cel) and ciltacabtagene autoleucel (cilta-cel). Other products have shown encouraging results in early-phase trials but remain investigational (e.g., ARI0002h, CC-98633/BMS-986354, anitocabtagene autoleucel, and equecabtagene autoleucel).

While CAR-T has demonstrated remarkable efficacy in relapsed/refractory multiple myeloma (RRMM), most patients eventually relapse. Retrospective studies have identified various factors that may affect clinical outcomes following T-cell redirecting therapies (TCRTs), including age, performance status, cytogenetic abnormalities, presence of extramedullary disease (EMD), prior treatments, and immunologic function, among others [3,4,5,6]. Early deaths from non-relapse mortality have also been observed after CAR-T administration, further underscoring the need to better understand and navigate the treatment process—from patient selection and leukapheresis to product infusion and toxicity management. For instance, practical considerations for bridging therapy selection should encompass both disease control through successful CAR-T manufacturing and preservation of T-cell fitness, with a particular focus on maintaining effector function and supporting a favorable tumor immune microenvironment.

Preclinical evidence suggests that selinexor—an oral selective inhibitor of nuclear export (SINE) compound targeting exportin 1 (XPO1)—may help mitigate T-cell exhaustion and potentially enhance outcomes from TCRTs [7,8]. Selinexor is currently approved for use in combination with bortezomib and dexamethasone for patients with RRMM who have received one or more prior therapy lines. Building on this foundation, we conducted an exploratory analysis to evaluate the impact of prior selinexor exposure on the clinical outcomes of patients undergoing BCMA-directed CAR-T for RRMM.

## 2. Materials and Methods

### 2.1. Study Design

This retrospective cohort study was conducted at two National Cancer Institute-designated cancer centers: Mount Sinai’s Tisch Cancer Institute (New York, NY, USA) and Cleveland Clinic’s Taussig Cancer Institute (Cleveland, OH, USA). The study adhered to the principles of the Declaration of Helsinki, with Institutional Review Board approval obtained at both centers and waivers of consent granted. Patients eligible for inclusion were required to be at least 18 years old, diagnosed with RRMM, and previously treated with a selinexor-based regimen prior to receiving autologous BCMA-directed CAR-T at either institution. Data were retrospectively collected through the review of electronic medical records.

### 2.2. Clinical Assessments

Disease status categories were determined using International Myeloma Working Group (IMWG) criteria [9] to calculate overall response rate (ORR), very good partial response or better rate (≥VGPR), and complete response rate (CRR) after CAR-T administration. Duration of response (DOR) was defined as the time from the first documented partial response (PR) or better to the earliest occurrence of either disease progression or death from any cause. Progression-free survival (PFS) was defined as the time from CAR-T infusion to either disease progression or death from any cause, whichever occurred first. Overall survival (OS) was defined as the time from CAR-T infusion to death from any cause or the last follow-up. PFS and OS analyses included patients with sufficient follow-up and information on status of events. Patients who did not experience a PFS or OS-limiting event were censored at the date of their last follow-up. Cytokine release syndrome (CRS) and immune effector cell-associated neurotoxicity syndrome (ICANS) were graded per American Society for Transplantation and Cellular Therapy (ASTCT) criteria [10], while hematologic toxicities were graded per Common Terminology Criteria for Adverse Events (CTCAE), version 5.0.

### 2.3. Statistical Analysis

Due to the hypothesis-generating approach of this study, no formal sample size calculation was performed. Patient and disease characteristics were summarized descriptively, with results presented as medians, means, or proportions, alongside variability measures such as 95% confidence intervals (CIs) and interquartile ranges (IQRs). Time-to-event plots for PFS, DOR, and OS were generated using the Kaplan–Meier method, with a censoring date of 1 December 2023. Multivariable Cox proportional hazards regression was employed to evaluate pre-CAR-T factors potentially associated with PFS and OS. Potential variables for model inclusion were first identified through univariable screening with a pre-set *p*-value threshold of 0.25. A backward elimination process, guided by the likelihood ratio test (*p* < 0.10 for retention), was subsequently used to refine the final set of independent variables. Given the exploratory nature and limited sample size of the present analysis, which aimed to identify the best-fitting model for factors associated with survival outcomes following CAR-T rather than to establish statistical significance, *p*-values are not reported. All statistical analyses were performed using Stata, release 16.0 (StataCorp LLC, College Station, TX, USA).

## 3. Results

### 3.1. Patient Characteristics and Treatment History

Forty-five patients who received BCMA-directed CAR-T after prior exposure to a selinexor-based regimen were identified and included, with a median age of 64 years (range 50–78) at CAR-T infusion. Among the study population, 55.6% of patients were female, 88.9% had an Eastern Cooperative Oncology Group performance status (ECOG-PS) of 0–1, and 40% exhibited high-risk cytogenetics—defined by the identification of t(4;14), t(14;16), t(14;20), 17p deletion, and/or 1q gain/amplification on fluorescence in situ hybridization testing. Moreover, all patients were triple-class exposed and 91.1% had undergone at least one autologous stem cell transplantation (ASCT), with a median interval of 52 months (IQR 9.6–165) between the last ASCT and CAR-T infusion. Detailed baseline characteristics, including additional demographic and clinical variables, are presented in Table 1.

The median therapy line number was 7 (range 4–15) for selinexor use and 9 (range 6–15) for CAR-T administration. While 20 patients (44.4%) received selinexor in the line of therapy preceding CAR-T, 11 (24.4%) received this SINE as part of a bridging regimen. The median duration of selinexor-based therapy and median interval from last selinexor dose to CAR-T infusion were 2.7 (range 0.7–11.3) and 3.9 (range 0.7–22) months, respectively. Selinexor was most often used in combination with bortezomib/dexamethasone (28.9%) or carfilzomib/dexamethasone (20%), while combinations with anti-CD38 monoclonal antibodies (mAbs) or immunomodulatory drugs (IMiDs) were rarely used. Notably, 75.6% of patients started selinexor at a dose ≤ 80 mg weekly and 24.4% at a dose ≥ 100 mg weekly (Table 2).

As shown in Table 3, the autologous BCMA-directed CAR-T products administered included ide-cel (60%), cilta-cel (35.6%), and CC-98633/BMS-986354 (4.4%). During the manufacturing period, 31 patients (68.9%) received bridging therapy (not limited to selinexor-based treatment), with a median duration of 21 days. All patients underwent lymphodepletion with fludarabine/cyclophosphamide shortly before CAR-T infusion.

### 3.2. Efficacy Outcomes

Based on each patient’s best response to CAR-T over the study period, ORR, ≥VGPR, and CRR were 88.9%, 80%, and 53.3%, respectively. The median time to best response was 1.8 months (IQR 0.8–4.5). Table 4 provides a detailed breakdown of rates across specific IMWG categories, as well as other clinical outcomes. At a median follow-up of 68 months, median DOR was 8.1 months (IQR 2.6–39), median PFS was 8.1 months (IQR 3.1–39.5), and median OS was 35.9 months (IQR 14.2–not reached [NR]).

**Figure 1:**
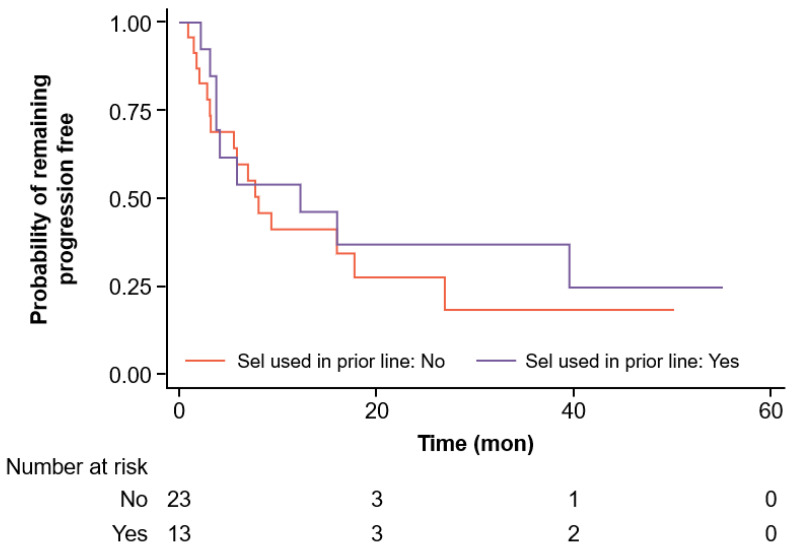
Progression-free survival if selinexor was used in the immediate prior line of therapy before CAR-T therapy.

A Cox proportional hazards regression analysis was performed to evaluate factors potentially associated with PFS and OS following BCMA-directed CAR-T for RRMM. As outlined in Table 5, our exploratory model for PFS retained five independent variables potentially influencing this outcome: (1) selinexor use in the immediate line of therapy preceding CAR-T; (2) ECOG-PS; (3) presence of EMD; (4) male sex; and (5) time interval from the last selinexor dose to CAR-T infusion. A trend emerged among patients receiving selinexor in the line of therapy immediately prior to CAR-T experiencing a 60% relative reduction in the risk of a PFS-limiting event, such as disease progression or death, compared with those exposed to selinexor in earlier lines (HR = 0.40; 95% CI 0.14–1.09; Figure 1).

**Figure 2:**
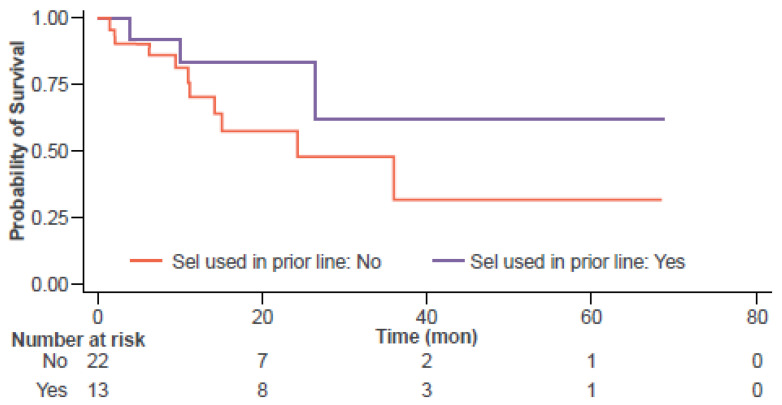
Overall survival if selinexor was used in the immediate prior line of therapy before CAR-T therapy.

In turn, our exploratory model for OS retained four independent variables potentially influencing this outcome: (1) selinexor use in the line of therapy immediately preceding CAR-T; (2) age ≥ 60 years; (3) pre-apheresis albumin levels; (4) male sex; and (5) time interval from the last selinexor dose to CAR-T infusion (Table 6). Consistent with the multivariable analysis for PFS, patients receiving selinexor in the line of therapy immediately prior to CAR-T exhibited a reduced risk of death (HR = 0.08; 95% CI 0.02–0.46; Figure 2). In this subgroup, median OS was NR at the 68-month follow-up. None of the 11 patients who underwent selinexor-based bridging died during follow-up, whereas all 13 patients who died after CAR-T had not received selinexor-based bridging.

### 3.3. Safety Outcomes

Table 4 summarizes hematologic parameters at baseline (pre-lymphodepletion), day 30, and day 100. CRS occurred in 34/45 patients (75.6%), with a median duration of 2 days and no grade ≥ 3 events. ICANS was reported in 8/45 patients (17.8%), with a median duration of 1 day. Among the ICANS cases, 2 were grade 3, while the remainder were grade 1 (n = 5) or grade 2 (n = 1). All affected patients fully recovered without any long-term sequelae.

## 4. Discussion

Recognizing the interplay of disease-, treatment-, and patient-related factors is critical to achieving the full benefits of CAR-T, given its high cost and typically one-time administration. An emerging strategy focuses on preserving T-cell fitness and optimizing the bone marrow microenvironment through careful treatment selection before leukapheresis and final product infusion. While certain agents used in MM management, such as alkylators and proteasome inhibitors (PIs), have been associated with inferior CAR-T outcomes [5], this exploratory analysis of patient-level data from two US centers suggests that prior selinexor exposure does not compromise the efficacy or safety of subsequent TCRTs. Selinexor use in our cohort, whether as part of a therapy line or as a bridging regimen prior to CAR-T, did not result in any detriments to expected ORR, DOR, PFS, OS, or incidence/severity of CAR-T-related toxicities [11]. Furthermore, patients who received selinexor in the therapy line immediately preceding CAR-T demonstrated longer PFS and OS compared to those exposed to this XPO1 inhibitor in earlier lines, with no deaths observed among those who underwent selinexor-based bridging prior to CAR-T infusion.

These findings align with earlier investigations suggesting selinexor-containing combinations as viable options for bridging regimens [12]. In a recent study evaluating the impact of bridging therapy on standard-of-care ide-cel outcomes, a median PFS of 9.8 months (95% CI 4.1–13.9) was reported for the 12% of patients (n = 26) who received selinexor-based bridging. Other combinations yielded varying PFS durations: 6.4 months (95% CI 2.70–12.50) for PI-based regimens, 6.5 months (95% CI 4.2–8.2) for alkylator-based regimens, and 12 months (95% CI 5.8–NR) for IMiD ± mAb-based regimens. In the overall cohort (n = 214), patients in the bridging group showed inferior OS compared to those in the non-bridging group (13.85 vs. NR months, *p* = 0.002), likely reflecting the higher tumor burden or more aggressive disease in those requiring bridging therapy to stabilize their condition before CAR-T infusion [13]. This observation may offer insight into a paradoxical finding from our study, where a shorter interval between last selinexor dose and CAR-T infusion was linked to inferior OS—a result that potentially reflects the use of selinexor in the bridging setting.

In the same retrospective study, the US Myeloma Immunotherapy Consortium reported a 38% incidence of grade ≥ 2 ICANS in selinexor-exposed patients. Despite the absence of patients with known central nervous system (CNS) pathology in their cohort, this finding may be attributed to selinexor’s ability to penetrate the blood–brain barrier, preexisting endothelial activation, and undetected CNS involvement from EMD [14,15]. By contrast, our cohort of 45 patients experienced grade ≥ 2 ICANS at a markedly lower rate of 6.7%. Supporting this observation, a case series of seven patients [16] with RRMM who received a selinexor-containing regimen immediately prior to apheresis for ide-cel or cilta-cel treatment, and a separate series of two patients with EMD [17] who received selinexor-based bridging and post-CAR-T maintenance, both reported no ICANS events. However, given the retrospective nature of these studies and the heterogeneity between study populations, confounding factors such as age, prior treatment history, and baseline disease characteristics may have influenced the differing rates of ICANS observed.

Translational research has shed light on the mechanisms by which selinexor modulates the tumor immune microenvironment. In a retrospective biomarker analysis of STOMP trial participants, Kang et al. conducted immuno-oncology profiling on paired bone marrow samples collected before and after selinexor-based treatment. Among CD3-positive cells, post-treatment samples showed higher expression of CD8 and granzyme B compared to screening samples, with the greatest increases observed when selinexor/dexamethasone was combined with pomalidomide over carfilzomib or daratumumab. Notably, T-cell inhibitory markers (such as PD-1, LAG3, and CTLA-4) were not induced in post-treatment samples [18]. Furthermore, XPO1 inhibition was found to promote the differentiation of myeloid-derived suppressor cells (MDSCs) into neutrophil-like immunostimulatory cells, thereby enhancing the antitumor reactivity of T cells [19]. Beyond direct effects on cytotoxic T-cell number and activity, selinexor has been associated with natural killer (NK) cell activation via disruption of the inhibitory NKG2A:HLA-E axis [20].

Using a mouse model of human non-Hodgkin’s lymphoma, Stadel et al. demonstrated a significant reduction in tumor burden when selinexor was administered prior to, rather than concurrently with, CD19-directed CAR-T [21]. This observation aligns with the previously discussed mechanisms, suggesting that selinexor may sensitize tumor cells to CAR-T-mediated cytotoxicity. Additionally, selinexor has been shown to upregulate BCMA expression in plasma cell lines, which could further enhance the efficacy of BCMA-targeted CAR-T for RRMM by increasing antigen availability for immune recognition and destruction [17].

Several limitations should be considered in this study. First, the patient sample was derived exclusively from two US academic centers, which may limit the generalizability of our findings, particularly across community-based practices and lower-resource settings. Second, the retrospective design of this non-randomized study introduces potential biases, including imbalances in baseline characteristics that could influence treatment selection and affect prognosis among patients who received selinexor-based regimens prior to CAR-T infusion. For instance, confounding by indication should be acknowledged when interpreting the role of selinexor as part of bridging therapy. Lastly, given the exploratory nature of this analysis, a formal sample size calculation was not undertaken, and consequently, *p*-values were not reported. Our primary objectives were to identify emerging trends rather than rigorously test specific hypotheses and to generate initial clinical insights rather than alter current management practices. As such, the present findings should be viewed as preliminary estimates intended to guide future research with larger cohorts, prospective designs controlling for treatments given prior to apheresis and as a bridge to CAR-T, and more robust statistical methods, ultimately leading to more conclusive results.

## 5. Conclusions

In conclusion, this exploratory observational study suggests that prior selinexor exposure does not compromise the efficacy or safety of anti-BCMA CAR-T in RRMM, with encouraging PFS and OS observed post-CAR-T in patients who were previously treated with selinexor-containing regimens. Preclinical data in the current literature support the hypothesis that XPO1 inhibition may reprogram the tumor immune microenvironment, making it less tumor-permissive. However, larger-scale, prospective studies are needed to assess whether selinexor use—either as part of the preceding therapy line or bridging regimens—can further enhance clinical outcomes from TCRTs. Additional preclinical research, including laboratory endpoints in future trials, will be essential to fully elucidating selinexor’s role in boosting antitumor immune responses and optimizing the T-cell microenvironment in RRMM, both before and after CAR-T infusion, as well as between distinct TCRT administrations.

## Figures and Tables

**Table 1:** Demographic and clinical characteristics of patient prior to the start of CAR-T therapy.

Parameter	n = 45
Median age at MM diagnosis (range)	54 (37–75)
Median age at the time of CAR-T therapy (range)	64 (50–78)
Female	55.6% (25)
Race	
White	75.6% (34)
Black	17.8% (8)
Other	6.7% (3)
ISS prior to CAR-T	
Stage I	31.1% (14)
Stage II	48.9% (22)
Stage III	11.1% (5)
Not Documented	8.9% (4)
ECOG Performance Status	
0 or 1	88.9% (40)
≥2	11.1% (5)
Cytogenetics	
t(4;14)	11.1% (5)
t(14;16)	4.4% (2)
del(17p)	15.6% (7)
1q21 gain/amp	55.6% (25)
High-risk cytogenetics abnormalities ^1^	40% (18)
Prior drug exposure	
lenalidomide	100% (45)
pomalidomide	97.8% (44)
bortezomib	97.8% (44)
carfilzomib	100% (45)
daratumumab	100% (45)
Prior ASCT	91.1% (41)
Second ASCT	31.1% (14)
Median time between last ASCT and CAR-T infusion (IQR)—months	52.0 (9.6–165)

Abbreviations: MM = multiple myeloma, ECOG: Eastern Oncology Cooperative Group, ISS = international stagging system, ASCT = autologous stem cell transplantation, IQR = interquartile range. ^1^ Presence of del(17p), t(4;14) and/or t(14;16) by fluorescence in situ hybridization.

**Table 2:** Characteristics of selinexor therapy prior to CAR-T therapy.

Parameter	n = 45
Selinexor regimen	
selinexor/bortezomib/dexamethasone	28.9% (13)
selinexor/daratumumab/carfilzomib/dexamethasone	4.4% (2)
selinexor/daratumumab/melphalan/prednisone	2.2% (1)
selinexor/dexamethasone	8.9% (4)
selinexor/bortezomib/isatuximab/dexamethasone	2.2% (1)
selinexor/isatuximab/dexamethasone	4.4% (2)
selinexor/carfilzomib	8.9% (4)
selinexor/carfilzomib/dexamethasone	20% (9)
selinexor/carfilzomib/venetoclax	2.2% (1)
selinexor/pomalidomide/dexamethasone	2.2% (1)
Not documented	15.6% (7)
Median number of LOTs (IQR)	7 (4–15)
Selinexor starting dose	
≤60 mg	13.3% (6)
70 mg	2.2% (1)
80 mg	60% (27)
100 mg	13.3% (6)
160 mg	11.1% (5)
Median duration of therapy (IQR)—months	2.7 (0.7–11.3)
Median duration of therapy when used in bridging (IQR)—months	2.6 (2–6.5)
Median duration of therapy when not used in bridging (IQR)—months	2.9 (0.7–10.8)
Median PFS with selinexor-based therapy (IQR)—months	2.3 (1–13.3)
Median time from last dose of selinexor to CAR-T infusion—months	3.9 (0.7–22)
Selinexor given as part of bridging regimen before CAR-T	24.4% (11)
Selinexor given as part of the LOT immediately preceding CAR-T	44.4% (20)

Abbreviations: IQR = interquartile range, PFS = progression free survival, LOT = line of therapy.

**Table 3:** Characteristics of CAR-T therapy delivered.

Parameter	n = 45
Product	
Idecabtagene vicleucel	60% (27)
Ciltacabtagene autoleucel	35.6% (16)
CC-98633/BMS-986354	4.4% (2)
Prior selinexor exposure	100% (45)
Median number of LOTs (IQR)	9 (6–15)
Bridging regimen administered	68.9% (31)
Median duration of bridging therapy (IQR)—days	21 (4–43)

Abbreviations: IQR = interquartile range, LOT = line of therapy.

**Table 4:** Clinical and toxicity outcomes following CAR-T.

Parameter	n = 45
Hematology Parameters (mean; std dev)	
Platelets at baseline—K/L	133 (84)
Platelets at day 30—K/L	65.3 (52.0)
Platelets at day 100—K/L	126 (74.9)
Hemoglobin at baseline—g/dL	10.0 (1.9)
Hemoglobin at day 30—g/dL	9.1 (1.9)
Hemoglobin at day 100—g/dL	10.7 (1.9)
Absolute neutrophil count at baseline—K/L	3.0 (1.9)
Absolute neutrophil count at day 30—K/L	1.3 (1.06)
Absolute neutrophil count at day 100—K/L	2.4 (1.2)
Best response to CAR-T therapy	
sCR	33.3% (15)
CR	20% (9)
VGPR	26.7% (12)
PR	8.9% (4)
SD	8.9% (4)
PD	2.2% (1)
Median time to best response (IQR)—months	1.8 (0.8–4.5)
Median duration of response (IQR)—months	8.1 (2.6–39)
CRS events (any grade)	75.6% (34)
Grade of CRS	
Grade 1	26
Grade 2	8
Median duration of CRS (IQR)—days	2 days (1–7)
ICANS events (any grade)	17.8% (8)
Grade of ICANS	
Grade 1	5
Grade 2	1
Grade 3	2
Median duration of ICANS (IQR)—days	1 (1–4)

Abbreviations: std dev= standard deviation, IQR = interquartile range, CR = complete response, PR = partial response, SD = stable disease, VGPR = very good partial response, sCR = stringent complete response, PD = progressive disease, CRS = cytokine release syndrome, ICANS = immune effector cell-associated neurotoxicity syndrome.

**Table 5:** Exploratory multivariable Cox regression analysis on PFS following CAR-T therapy.

Variable ^1^	Hazard Ratio ^2^	(95% CI)	Impact on Risk of Progression
Selinexor used in line immediately prior to CAR-T	0.40	(0.14–1.09)	↓ by 60%
ECOG PS: ≥1 vs. 0	2.30	(0.88–6.10)	↑ 2.3 times
EMD present prior to CAR-T therapy	3.54	(1.26–9.92)	↑ 3.5 times
Male gender	0.46	(0.19–1.12)	↓ by 54%
Time from the last dose of selinexor to CAR-T (mon)	0.94	(0.89–1.00)	↓ by 6% for each additional month

Abbreviations: PFS = progression-free survival, EMD = extramedullary disease, ECOG PS = Eastern Cooperative Oncology Group performance status. ^1^ The intent of this exploratory analysis was to identify the best-fitting model in terms of factors associated with PFS following CAR-T therapy. The intent was not to determine statistical significance, as the sample size was limited. ^2^ An HR of less than one indicates a lower risk and greater than one an increased risk of disease progression.

**Table 6:** Exploratory multivariable Cox regression analysis on OS following CAR-T therapy.

Variable ^1^	Hazard Ratio ^2^	(95% CI)	Impact on Risk of Death
Selinexor used in line immediately prior to CAR-T	0.08	(0.02–0.46)	↓ by 92%
Age ≥ 60 years	3.83	(0.94–15.5)	↑ 3.8 times
Albumin level (g/dL) prior to CAR-T	0.16	(0.04–0.62)	↓ by 84% risk with higher levels
Time from the last dose of selinexor to CAR-T (mon)	0.86	(0.74–0.98)	↓ by 14% for each additional month

^1^ The intent of this exploratory analysis was to identify the best-fitting model in terms of factors associated with OS following CAR-T therapy. The intent was not to determine statistical significance, as the sample size was limited. ^2^ An HR of less than one indicates a lower risk and greater than one an increased risk of death.

## Data Availability

The data presented in this study are available on request from the corresponding author. The data are not publicly available due to privacy restrictions.
